# Longitudinal Impact of Agitation and Aggression Symptoms on Societal Costs in Alzheimer's Disease: A Focus on Informal Care and Socioeconomic Disparities

**DOI:** 10.1002/gps.70257

**Published:** 2026-10-01

**Authors:** Nadège Costa, Célya Marcélo Fleuret, Eugénie Gombault, Maria Soto Martin, Adélaïde De Mauleon

**Affiliations:** ^1^ Health Economic Unit Toulouse University Hospital Toulouse France; ^2^ CERPOP Inserm Toulouse France; ^3^ University of Toulouse III UPS Toulouse France; ^4^ Department of Geriatric Medicine Toulouse University Hospital Toulouse France

**Keywords:** agitation/aggression, dementia, health care costs, informal care, longitudinal k‐means clustering, socioeconomic factors

## Abstract

**Background:**

To date, no longitudinal study has assessed the effect of agitation on societal costs—particularly informal care costs—over time in Alzheimer's Disease (AD) patients with Agitation and Aggression (A/A) symptoms.

**Objectives:**

This study estimates the impact of A/A symptoms evolution on longitudinal care costs including informal care costs in moderate‐to‐severe AD, considering patient and caregiver socioeconomic characteristics.

**Design:**

A longitudinal trajectory clustering method was used on this longitudinal, prospective, multicenter observational cohort study. Setting and Participants: 262 community‐dwelling and long‐term care facility (LTCF) patients of both sexes, aged 60 years and older, with clinically significant A/A symptoms were recruited from memory centers in southwest France.

**Methods:**

Patients were followed for 12 months. A/A data was recorded by clinicians using the Neuropsychiatric Inventory (NPI‐c). Costs were estimated from a societal perspective, including healthcare and informal care, using the RUD questionnaire. K‐means longitudinal method identified A/A evolution profiles. Cost differences across A/A evolution profiles were analyzed using Generalized Linear Models.

**Results:**

Two A/A evolution profiles were identified: mild symptoms at baseline with improvement (A) and moderate symptoms at baseline with limited improvement or stability (B). Annual costs were €22,959 in group A and €23,625 in group B excluding supervision, and €52,111 and €56,149, respectively, including supervision. In LTCF residents, higher A/A severity was associated with increased medical visits and medication costs, though overall cost differences were smaller. Informal care neared 70% of total costs for community‐dwelling patients, with supervision covering the majority of these costs.

**Conclusions:**

Evolution of A/A symptoms significantly increases both healthcare and informal care costs, particularly in at‐home settings. Integrating systematic monitoring of behavioral symptoms into routine care would enable personalized, cost‐effective strategies. Informal care costs, often undervalued and overlooked in economic analyzes, underscores the need for improvement in resource allocation and quality of life, for both patients and caregivers.

**Clinical trial number:**

NCT03554226

## Key Points

- Greater Agitation and Aggression (A/A) symptom severity was associated with higher annual healthcare costs in community‐dwelling patients with Alzheimer’s Disease (AD).
- Moderate A/A was associated with a 22% increase in annual healthcare costs after adjustment for age, sex, and ADL score.
- Informal care represented a substantial proportion of total costs among community‐dwelling patients, nearly 70%, largely driven by supervision.
- Longitudinal trajectories of A/A symptoms differed between community‐dwelling and long‐term care facility patients, with distinct patterns of symptom evolution across care settings.

## 1 Introduction

According to the World Alzheimer's Report, over 55 million people worldwide live with dementia—a figure likely underestimated due to the underdiagnosis of Alzheimer's disease (AD). The global societal cost of AD is estimated at US$1.3 trillion, with 85% of these costs borne by families or social care systems [1]. Among the most burdensome neuropsychiatric symptoms (NPS) are agitation and aggression (A/A), characterized by verbal or physical aggressiveness and excessive motor activity [2]. These symptoms affect 30%–50% of AD patients and are strongly associated with caregiver burden, risk of institutionalization, and increasing societal costs [3, 4].

A 2015 UK study estimated that agitation symptoms lead to a £4091 annual increase in costs per AD patient [5]. However, that study did not include informal care, which accounts for the largest share of total societal costs. In a previous European analysis, our team found that 73% of the additional costs attributable to agitation were linked to informal care [6].

The incidence and consequences of A/A symptoms may be influenced by socioeconomic status. A 2018 UK study [7] found that wealth predicts dementia onset, while other research has shown that caregiver characteristics such as younger age, low education, depression, and perceived burden increase the risk of NPS in patients [8]. Disadvantaged women with NPS are also more likely to be prescribed antidepressants and institutionalized sooner [9].

To date, no longitudinal study has assessed the effect of agitation on societal costs—particularly informal care costs—over time. Additionally, the role of socioeconomic characteristics of patients and caregivers on care costs in AD patients with A/A symptoms has not been studied.

This study aims to assess the longitudinal impact of agitation on total care costs among home‐dwelling and institutionalized AD patients, with a particular focus on the influence of socioeconomic characteristics on costs and quality of life.

## 2 Methods

### 2.1 Population, Setting and Design

This study uses data from the A3C cohort (Agitation and Aggressiveness in Alzheimer's disease), a prospective multicenter observational study carried out in southwestern France [10]. The cohort aimed to examine the natural course of clinically significant A/A symptoms over a 12‐month follow‐up. Patients aged ≥ 60 years, diagnosed with possible AD per NIA‐AA criteria, and living either at home or in Long‐Term Care Facilities (LTCFs) were recruited.

Clinically significant agitation was defined by at least one of the following NPS from the Neuropsychiatric Inventory (NPI): aggression, disinhibition, aberrant motor behavior, or irritability. A total of 262 patients were enrolled.

### 2.2 Agitation and Aggression Symptoms

A/A severity was assessed at baseline and follow‐up using the NPI‐clinician version (NPIc), collected by trained professionals through face‐to‐face interviews [11].

### 2.3 Cost Estimates

Healthcare and informal care resource use was assessed via the Resource Utilization in Dementia (RUD) questionnaire, capturing medical visits, hospital stays, medications, paramedical services, and informal care including basic (BADL), instrumental (IADL), and supervisory care. Supporting Information S1: e‐Tables 1 and 2 in appendix details how healthcare costs were valued.

Valuation followed a societal perspective; Hospital stays: Valued using average DRG tariffs [12]. Medical/paramedical care: Valued using French National Health Insurance (FNIH) tariffs and claims databases [13]. Medications: Priced based on French tariffs and prescribed packaging [14]. Informal care: Valued via the proxy good method [15] using the wage of a care assistant (€12.77/hour) [16]. LTCF costs: Estimated at €123.82/day, based on national rates for care, dependency, and accommodation [17].

### 2.4 Covariates

Socioeconomic data (education, occupation, marital status) of patients and caregivers were recorded, along with living arrangement, caregiver‐patient relationship, comorbidities (Charlson Comorbidity Index) [18], and AD duration [10].

### 2.5 Statistical Analyses

We used a longitudinal trajectory clustering method to group similar trajectories of A/A evolution during the 1‐year follow‐up period. K‐Means Longitudinal (KmL) method, a hill climbing clustering algorithm, was used to investigate longitudinal trajectories of NPI‐c A/A [19]. Costs were not used to define the clusters as cluster assignment was based exclusively on the longitudinal trajectories of NPIc A/A symptoms. These trajectories were partitioned into *k* sets while minimizing the distance between trajectories within each partition using the Euclidean distance. The selection of the optimal number of clusters was based upon a combination of quality criteria: the Calinski and Harabasz score (CH), Bayes Information Criteria (BIC), and Akaike Information Criteria (AIC) [20]. Trajectories with one or two missing data were imputed with CopyMean method.

GRU‐D was used to address missing caregiver‐reported informal care time collected through the Resource Utilization in Dementia (RUD) questionnaire. Some estimates were missing at individual follow‐up assessments. A standard and relatively simple GRU‐D architecture was used to estimate missing values while accounting for the longitudinal structure of the data; imputations were generally close to the mean. Observed values were retained unchanged [21].

Baseline characteristics of the whole population and of each cluster for each care setting are presented in percentages and means (standard deviation) as appropriate. Chi square tests and Mann Whitney non‐parametric tests were used to compare characteristics between clusters. Annual cost of care was analyzed using mean and Bias‐Corrected and accelerated bootstrap 95% Confidence Intervals (CI). Mann Whitney non‐parametric test was used to compare costs between clusters.

Generalized Linear Model (GLM) with gamma distribution and log link function was used to examine the associations of different A/A evolution profiles with cost of care [22]. The explicative covariate of interest was the A/A evolution, and other clinical and demographic covariates were included.

Statistical analyses were implemented using the *R* software, version 4.1.1.

## 3 Results

The final analysis included 183 community‐dwelling (CD) patients and 79 in LTCFs.

Figure 1 shows that for each care setting, community and LTCF, two distinct trajectories of A/A symptom severity were identified using longitudinal clustering.

**FIGURE 1:**
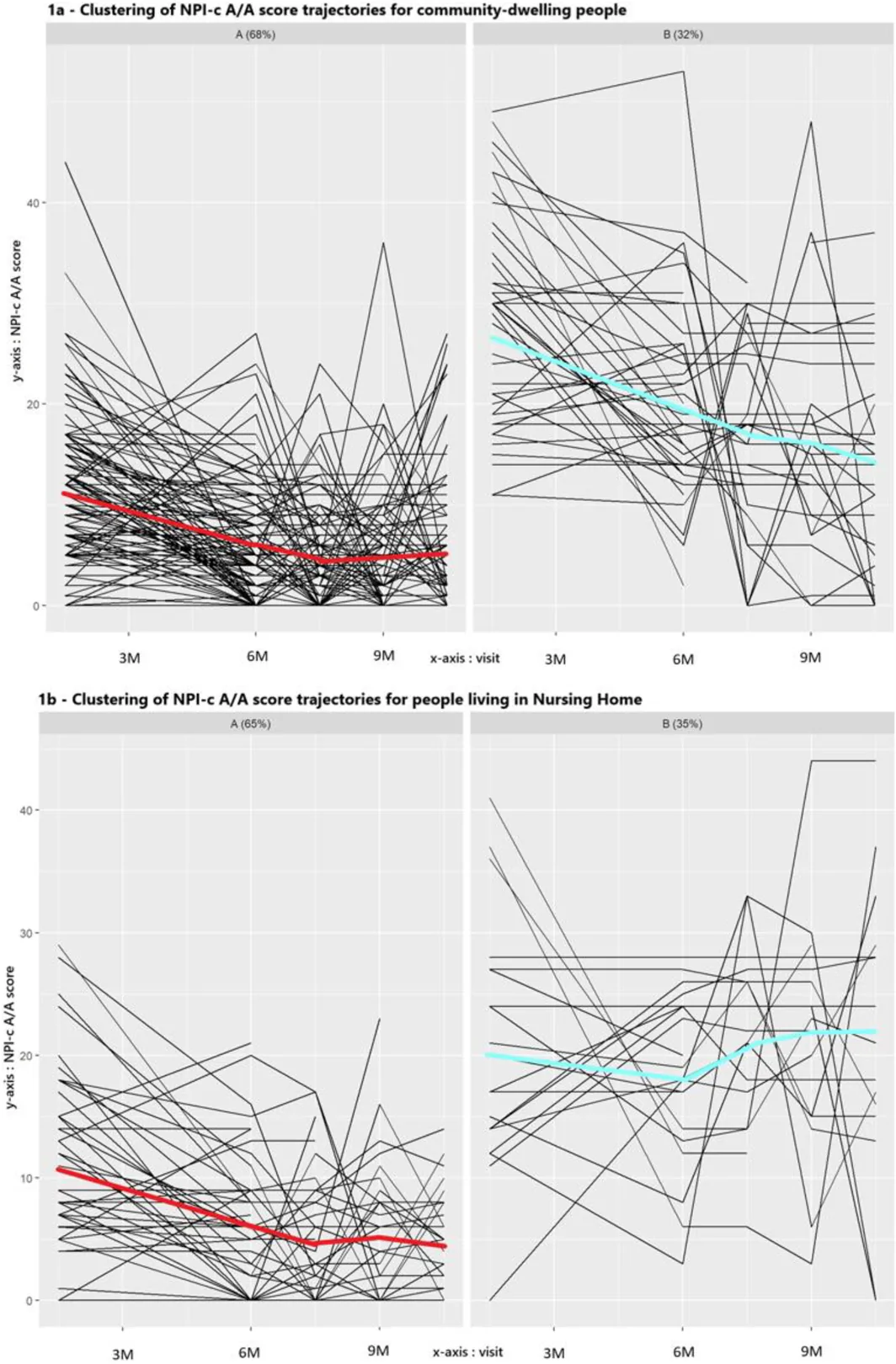
Clustering of NPI‐c A/A score trajectories.

For CD individuals:

Group A included patients with mild A/A symptoms at baseline who showed an overall improvement during follow‐up.

Group B included patients with moderate A/A symptoms at baseline experiencing almost no improvement during follow‐up.

For LTCF individuals:

Group A included residents with mild A/A symptoms at baseline who showed clinical improvement during follow‐up.

Group B included residents with moderate A/A symptoms at baseline whose symptom severity remained stable or worsened during follow‐up.

Table 1 presents the baseline characteristics of patients according to these A/A symptom trajectories within each care setting. Overall, no significant differences were observed between groups within each setting. Women were overrepresented in both settings, with a mean age of 81 years for CD patients and 85 years for LTCF residents. Most patients had attained tertiary education and had no major comorbidities. Functionally, most CD individuals presented moderate dependency, while LTCF residents were primarily classified as severely dependent. Table 2 presents annual costs of care according to group and care setting. For CD, a non‐significant trend toward higher annual total costs was observed in group B compared with group A (*p* = 0.43). Annual total costs rise from €22,959 (Group A) to €23,625 (Group B) excluding supervision costs and from €52,111 (Group A) to €56,149 (Group B), supervision costs included. Cost increase between group A and B is mainly led by informal care costs and specifically by help for ADL that increase non‐significantly by 44% (*p* = 0.08). Formal care costs showed a non‐significant 46% decrease between group B and A (*p* = 0.08). Paramedical care costs were €940 higher on average in Group A than in Group B, although this difference was not statistically significant (*p* = 0.29). Costs specifically related to physiotherapy and occupational therapy were significantly higher in Group A, with a mean difference of €239 (*p* = 0.04). The formal care cost decreased between group B and A was mainly led by home care. For LTCF patients we observed an increase of annual total cost for patients in group B in comparison with group A. Annual total costs increased from €54,825 for group A to €55,680. This difference was not statistically significant. Cost increase was mainly driven by a 52% increase in medical visits (*p* = 0.01), while medication costs showed a non‐significant 25% increase (*p* = 0.12).

**TABLE 1:** Patient's characteristics according to clusters and place of living.

	Community‐dwelling people			Nursing home people		
Baseline characteristics	Group A	Group B	*p*‐value	Group A	Group B	*p*‐value
*N*	139	44		56	23	
Sex patient, female (*N*, %)	80 (57.6%)	26 (59.1%)	> 0.9	35 (62.5%)	12 (52.2%)	0.81
Age patient, mean (SD)	81.4 (6.7)	80.8 (6.0)	0.61	85. 1 (7.5)	84.9 (9.3)	0.61
Education—patient (*N*, %)			0.82			0.79
Tertiary education or lesser	85 (61.2%)	28 (63.6%)		35 (62.5%)	16 (69.6%)	
High school diploma or equivalent	28 (20.1%)	9 (20.5%)		12 (21.4%)	5 (21.7%)	
Short and long cycles higher education	26 (18.7%)	7 (15.9%)		9 (16.1%)	2 (8.7%)	
Patient's occupation before retirement (*N*, %)			0.53			0.21
Farmer	3 (2.2%)	2 (4.5%)		2 (3.6%)	4 (17.4%)	
Craftsmen and retailers	21 (15.1%)	3 (6.8%)		7 (12.5%)	4 (17.4%)	
Senior executive and intellectual profession	22 (15.8%)	3 (6.8%)		7 (12.5%)	4 (17.4%)	
Intermediate profession	25 (18.0%)	9 (20.5%)		6 (10.7%)	2 (8.7%)	
Employee	34 (24.5%)	17 (38.6%)		17 (30.4%)	4 (17.4%)	
Worker	19 (13.7%)	5 (11.4%)		5 (8.9%)	3 (13.0%)	
NA	15 (10.8%)	5 (11.4%)		12 (21.4%)	2 (8.7%)	
Charlson comorbidity index (*N*, %)			0.32			0.19
0	85 (61.2%)	31 (70.5%)		31 (55.4%)	10 (43.5%)	
1–2	46 (33.1%)	10 (22.7%)		20 (35.7%)	8 (34.8%)	
> 2	8 (5.8%)	3 (6.8%)		5 (8.9%)	5 (21.7%)	
Lifestyle—patient (*N*, %)			0.85			
At home—alone	22 (15.8%)	7 (15.9%)		/		
At home—with partner	91 (65.5%)	30 (68.2%)		/		
At home—with someone ≠ partner	26 (18.7%)	7 (15.9%)		/		
ADL at baseline (*N*, %)			0.29			0.78
[0–2] (dependency)	13 (9.4%)	4 (9.1%)		30 (53.6%)	12 (52.2%)	
[3–4] (moderate dependency)	44 (31.7%)	11 (54.5%)		10 (17.9%)	4 (17.4%)	
> 4 (mild or no dependency)	56 (40.3%)	24 (25.0%)		4 (7.1%)	1 (4.3%)	
NA	26 (18.7%)	5 (11.4%)		12 (21.4%)	6 (26.1%)	
MMSE at baseline (*N*, %)			0.65			0.14
> 22 (mild to none)	17 (12.2%)	6 (13.6%)		1 (1.8%)	1 (13.6%)	
[11–21] (moderate)	64 (46.0%)	23 (52.3%)		15 (26.8%)	3 (52.3%)	
< 10 (severe)	55 (39.6%)	15 (34.1%)		38 (67.9%)	17 (34.1%)	
NA	3 (2.2%)	/		2 (3.6%)	2 (8.7%)	
Patient's marital status, married (*N*,%)	94 (67.6%)	29 (65.9%)	> 0.9	20 (35.7%)	9 (39.1%)	> 0.9

**TABLE 2:** Annual costs by groups according to place of living.

	Community‐dwelling people (*N* = 183)			Nursing home people		
Annual costs	Group A (*N* = 139)	Group B	*p*‐value	Group A	Group B	*p*‐value
	Mean [95% CI]	Mean [95% CI]		Mean [95% CI]	Mean [95% CI]	
Inpatient stays	**5189 [4156–6222]**	**4153 [2883–5423]**	**0.20**	**3903 [2077–5729]**	**4163 [2122–6204]**	**0.32**
Medical visists	**466 [395–538]**	**526 [381–672]**	**0.46**	**363 [279–447]**	**802 [525–1080]**	**< 0.01**
Paramedical acts	**4742 [3655–5829]**	**3802 [2394–5210]**	**0.29**	**561 [241–882]**	**544 [101–987]**	**> 0.90**
Nurse	4092 [3090–5094]	3392 [2028–4755]	0.40	—	—	—
Physiotherapist and occupational therapy	649 [476–823]	410 [244–576]	0.04	561 [241–882]	544 [101–987]	> 0.90
Medication	**2189 [2027–2351]**	**2258 [1935–2582]**	**0.68**	**1604 [1204–2003]**	**2143 [1725–2561]**	**0.12**
Formal care	**3298 [1809–4787]**	**1782 [937–2628]**	**0.08**	—	—	—
Home care	2371 [955–3786]	955 [368–1543]	0.06	—	—	—
Day care	320 [142–499]	487 [102–869]	0.43	—	—	—
Meals on wheels	577 [239–915]	304 [42–649]	0.26	—	—	—
Others	30 [13–46]	38 [6–70]	0.63	—	—	—
Informal care	**36,726 [32,814–40,639]**	**44,080 [35,332–52,829]**	**0.12**	—	—	—
Help for BADL	3674 [3066–4462]	6554 [3454–9653]	0.08	—	—	—
Help for IADL	3810 [3275–4344]	5003 [3149–6857]	0.21	—	—	—
Supervision	29,152 [25,751–32,552]	32,523 [26,159–38,888]	0.35	—	—	—
Nursing home	—	—	—	48,408	48,048	—
*Total costs w/o supervision*	***22,959 [20,045*–*25,873]***	***23,625 [18,494*–*28,755]***	***0.82***	—	—	—
Total costs	**52,111 [47,117–57,105]**	**56,149 [47,049–65,248]**	**0.43**	**54,825 [52,910–56,740]**	**55,680 [53,617–57,744]**	**0.53**

*Note* *:* Bold values were used solely to highlight the main cost categories and total costs.

Figure 2 shows the evolution of total annual costs every 4 months. For community dwelling patients this figure shows that cost globally decreased between baseline and twelve months for group A while they increased for group B. For LTCF patients, total annual costs decreased for both groups between baseline and twelve months, following the same pattern.

**FIGURE 2:**
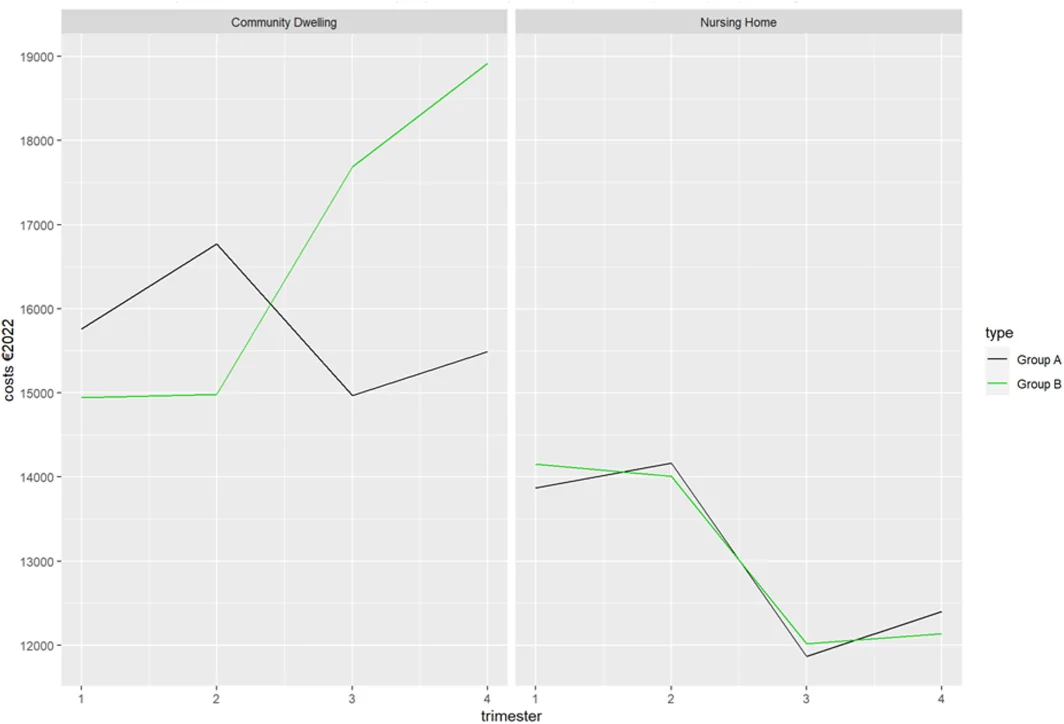
Annual total costs evolution according to groups and place of living.

Results on costs predictors are summarized in Table 3. When total costs were adjusted on age and sex (Model 1), we could observe that moderate A/A and moderate severity of dementia had no impact on total annual healthcare costs regardless of care setting. Only severity of dementia increased significantly healthcare costs of CD patients by 52% (*p* = 0.003). When total annual costs were adjusted on age, sex and ADL score, the model showed that moderate A/A symptom severity increased total annual costs by 22% (*p* = 0.04) compared to mild A/A symptom severity in community dwelling patients.

**TABLE 3:** Total annual costs predictors.

Cost Ratio/exp (coef), 95% CI, value														
Community‐dwelling people				Nursing home people					Community dwelling people			Nursing home people		
Adjusted [$1]									Adjusted [$2]					
NPI‐c A/A clusters	Grp A	Ref	—		Ref	—			Ref	—		Ref	—	
	Grp B	1.08	0.90, 1.32	0.4	1.02	0.96, 1.08		0.5	**1.22**	**1.00, 1.48**	**0.04**	1.04	0.98, 1.11	0.2
MMSE baseline	Mild to none	Ref	—		Ref	—			Ref	—		Ref	—	
	Moderate	1.21	0.92, 1.58	0.1	1.11	0.94, 1.31		0.2	1.12	0.86, 1.46	0.4	1.16	0.99, 1.36	0.06
	Severe	**1.52**	**1.15, 1.97**	**0.003**	1.02	0.86, 1.19		0.8	1.30	0.98, 1.70	0.07	1.05	0.90, 1.21	0.5
Modified CCI	0								Ref	—		Ref	—	
	1–2								0.90	0.74, 1.09	0.3	0.95	0.90, 1.01	0.11
	> 2								*0.69*	*0.46, 1.08*	*0.08*	0.99	0.91, 1.09	0.9

*Note:* [$1] Age [65–78], [79–84], [85–95], Sex. [$2] Age [65–78], [79–84], [85–95], Sex, ADL score at baseline [dependent < 3; moderate 3–4; independent > 4].

## 4 Discussion

Our study indicates that the annual progression in the severity of agitation and aggression (A/A) symptoms significantly influences both healthcare and informal care costs, particularly among CD older adults with Alzheimer's disease (AD). Across both care settings, greater symptom progression is consistently associated with higher costs.

We considered it essential to focus on individual longitudinal trajectories of informal care needs rather than cross‐sectional “point‐in‐time” data because the progression of A/A symptoms is highly heterogeneous across individuals. A longitudinal approach provides a nuanced understanding of symptom evolution. In contrast, point‐in‐time data offer only a static snapshot, failing to capture the dynamic and variable nature of these symptoms over time. A/A symptoms are associated with increased healthcare costs. A 2021 study conducted in the United States across both care settings found that A/A symptoms led to an annual increase of $10,798 in direct healthcare costs. This rise was primarily driven by professional caregiver expenses for CD individuals, and by long‐term care facility (LTCF) costs for residents living in LTCFs [23]. The severity of A/A symptoms was associated with increased healthcare costs. Although the study recorded the number of informal care hours, it did not report associated informal care costs. It found that individuals with dementia and no agitation received an average of 33 hours of informal care per week, compared to 48 hours per week for those exhibiting agitation.

In our study, informal care accounted for approximately 70% of total annual costs among CD individuals with AD. Supervision alone represented nearly 80% of these informal care costs, highlighting its substantial contribution. To ensure a realistic estimation of daily informal care time, caregivers were asked to report their average hours of sleep, thereby restricting the caregiving period to waking hours. On average, caregivers reported 7 hours of sleep per night, limiting the potential caregiving time to a maximum of 17 hours per day.

Importantly, supervision is an intangible activity that lacks a concrete or measurable output—unlike tasks such as meal preparation—and is therefore often undervalued or overlooked in economic analyses. Our findings emphasize the need to better recognize and account for the hidden burden of supervisory care in cost assessments [24]. The potential overestimation of supervision time may partly be explained by joint production, where caregiving activities are performed alongside routine tasks, as well as by the tendency to report normal household chores as care‐related duties in response to caregiving demands. These factors can blur the boundary between care‐specific and general domestic activities, leading to inflated estimates of supervision time [25]. Nevertheless, some studies show more supervision time for patients with dementia than for patients with other conditions and for informal caregiver who lives with patient with dementia [24]. In our study, around 70% of caregivers live with care recipient that demonstrated the high level of supervision time.

Caregivers play a pivotal role in supporting the health and well‐being of older adults who require ongoing assistance. In the United Kingdom, they are considered the largest provider of healthcare services, with an estimated economic value ranging from US$77 to US$182 billion annually. This underscores the essential contribution of informal caregivers to the sustainability of health and social care systems [24]. In France, nursing homes are financed through three main channels. The “care” budget is fully covered by the Regional Health Agency via national health insurance and primarily supports medical staff and equipment. The “accommodation” budget, which includes housing, meals, and recreational activities, is paid by residents, with potential financial assistance depending on their income. The“dependency” budget is mainly funded by local departments and partially by residents through the Personal Autonomy Allowance (Allocation Personnalisée d’Autonomie, APA). This budget covers support with activities of daily living and could conceptually be compared to the economic value of informal care provided in the community setting [6, 26]. As the European population continues to age, there is an increasing demand for alternatives to publicly funded long‐term care. In response, many Western European countries are undertaking reforms of their long‐term care systems, emphasizing civic engagement, self‐management, and mutual support within communities [27]. The ongoing caregiver crisis represents a major challenge: the combination of an aging population and the high burden placed on informal caregivers is contributing to a growing shortage of available caregiving resources. This shortage has direct implications for the quality and accessibility of care, particularly for the most vulnerable individuals who rely heavily on informal support [28]. In response, there is growing recognition of the urgent need to support caregivers with sustainable, cost‐effective, and preventive solutions. These approaches aim to reduce caregiver burden and ensure quality, continuous care.

In CD patients, physiotherapists and occupational therapists were included under the category of paramedical care, as they make up the Alzheimer's Specialized Teams (Équipes Spécialisées Alzheimer, ESA) [29].

These multidisciplinary teams are tasked with supporting CD individuals diagnosed with AD and strengthening the patient‐caregiver relationship. Supervised by coordinating nurses, ESAs include dementia‐trained professionals, primarily occupational therapists and psychomotor therapists. In CD patients, paramedical care, particularly physiotherapy and occupational therapy provided through Alzheimer's Specialized Teams (Équipes Spécialisées Alzheimer, ESA), was more frequently used in Group A. These multidisciplinary interventions aim to improve and, importantly, preserve autonomy in activities of daily living (ADLs). This may be consistent with the higher proportion of patients with mild or no dependency at baseline in Group A. This is consistent with the baseline characteristics of the study population, as 40.3% of individuals in Group A had mild or no dependency at study entry, compared to only 25% in Group B.

In our study, home care costs were €1516 lower in Group B than in Group A. This reduction may partially explain the higher informal care costs observed in Group A. One reading is that, prior to institutionalization, patients in Group A may have exhibited resistance to professional care, thereby a greater burden is shifted on informal caregivers. Previous studies have shown that increased caregiver burden is closely associated with disruptive behaviors such as aggression, screaming, and low functional ability in activities of daily living (ADLs) [30].

In day care settings, patients are typically offered cognitive and/or functional workshops. However, behavioral symptoms like A/A may limit access to these programs, delegating care back to informal caregivers. This pattern is reflected in our findings, showing higher informal care costs in Group B (€44,080) than Group A (€36,726) although without statistical significance (*p* = 0.12). When behavioral symptoms become too severe, traditional day care programs are no longer appropriate. In such cases, patients may be referred to specialized “Drop‐In” programs offering suitable supervision and activities. Access to these services is often limited by geographic gaps and low awareness among caregivers and providers, leading to care discontinuity and more reliance on informal support. In both care settings, patients in Group B were prescribed a greater number of medications. At the time of the study, there was an increasing trend in the use of psychotropic drugs and anti‐Alzheimer's medications, both of which contributed to the management of A/A symptoms. Health professionals tended to maintain anticholinesterase inhibitors despite disease progression, prioritizing their role in managing behavior and limiting psychotropic medication use, aimed at optimizing patient care, even if costly [31].

Our study has several limitations. First, agitation severity was assessed using the A/A domain from the clinician‐rated Neuropsychiatric Inventory (NPI‐c). Although the NPI‐c‐IPA (Neuropsychiatric Inventory—Clinician version with additional Instrumental Activities of Daily Living items) provides complementary information on the impact of symptoms on instrumental activities of daily living, we opted for the NPI‐c as it focuses exclusively on neuropsychiatric symptoms, while data on activities of daily living were independently available in our dataset [32]. Second, participants were recruited from Memory Clinics, which may limit the generalizability of our findings to the broader population of individuals with AD, as healthcare utilization and care needs may differ in less selected populations. Third, we were unable to analyze the impact of A/A symptom profiles among LTCF residents due to the limited sample size in this subgroup. We did not have access to individual‐level real charges for LTCF residents. While missing overall resident charges in other settings were imputed using deep learning methods, this was not possible for LTCF charges, as our database did not contain this information. However, missing ADL informal care data had limited relevance for LTCF residents, as ADL care is generally provided within the institution. Instead, we estimated average LTCF costs based on national flat‐rate components for dependency, care, and accommodation. Using this method, the estimated annual cost per resident with dementia ranged from €67,160 to €68,620 [33]. We were not able to collect detailed individual‐level cost data for LTCF residents. In general, economic studies conducted in LTCFs remain scarce, with most existing research focusing on CD individuals and adopting either a healthcare payer or patient perspective. This limits the comparability of our findings with the broader literature. Integrating real LTCF expenditure data in future research would provide a more accurate understanding of the specific burden associated with A/A symptoms in institutional settings. It would also support the optimal allocation and planning of human resources within LTCFs to improve the quality of care provided to residents.

### 4.1 Conclusion and Implication

To date, few studies have specifically examined the economic impact of agitation and aggression (A/A) symptoms in Alzheimer's disease (AD) across both home and institutional settings, on moderate‐to‐severe AD patients. Our study contributes to filling this gap by providing longitudinal cost estimates linked to the progression of A/A symptoms, using real‐world data and a data‐driven approach to patient profiling. Our findings suggest that greater A/A severity may be associated with higher healthcare and informal care costs, particularly among community‐dwelling individuals. These findings highlight the central role of informal caregivers, whose support—often invisible in cost evaluations—represents a substantial portion of the overall care burden. In parallel, the results stress the importance of early interventions and adapted professional support, such as the Alzheimer's Specialized Teams, to maintain patient autonomy and delay institutionalization. Finally, integrating systematic monitoring of behavioral symptoms and intrinsic capacity into routine care pathways would allow for more personalized and cost‐effective strategies, especially in nursing homes. Such approaches could guide resource allocation and improve the quality of life for patients and their caregivers as they are a key pillar in the care process.

## Funding

The A3C cohort was supported by Ethypharm and Toulouse University Hospital.

## Ethics Statement

The study was conducted in accordance with the Declaration of Helsinki and its later amendments or comparable ethical standards. All methods were carried out in accordance with relevant guidelines and regulations (MR004). All experimental protocols were approved by the Commission Nationale de l’Informatique et des Libertés.

## Consent

All patients included in the A3C cohort have signed a written agreement, in accordance with the agreements of the National Data Protection Commission. Hence, informed consent was obtained for all subjects and/or their legal guardian(s) for their data to be used to research ends and for publication.

## Conflicts of Interest

The authors declare no conflicts of interest.

## Supporting information


Supporting Information S1

## Data Availability

The data that support the findings of this study are available on request from the corresponding author. The data are not publicly available due to privacy or ethical restrictions.
