# Supplementary material for: Longitudinal Impact of Agitation and Aggression Symptoms on Societal Costs in Alzheimer's Disease: A Focus on Informal Care and Socioeconomic Disparities
Source: Int J Geriatr Psychiatry. 2026 Oct 1;41(10):e70257. doi: 10.1002/gps.70257 (PMC13630399; doi:10.1002/gps.70257)
Supplement: Supplementary file 1 [file GPS-41-e70257-s001.pdf]

Supplementary material

e-Table 1: Trimestral €2022 costs according to the place of living of the patient and to the NPI-C clusters Community dwelling patients, n = 183

		Mean costs per patient T1<br>n = 157			Mean costs per patient T2<br>n = 146			Mean costs per patient T3<br>n = 137			Mean costs per patient T4<br>n = 125		
		**Group A**<br>**n = 113**	**Group B**<br>**n = 44**	*p*	**Group A**<br>**n = 109**	**Group B**<br>**n = 37**	*p*	**Group A**<br>**n = 104**	**Group B**<br>**n = 33**	*p*	**Group A**<br>**n = 92**	**Group B**<br>**n = 33**	*p*
**Informal Care**<br>[IC 95%]<br>*Nbr H µ*	**ADL**	**954€**<br>[677 – 1,231]<br>*89*	**1,487€**<br>[906 – 2,068]<br>*138*	*0,1*	**1,175€**<br>[846 – 1,504]<br>*109*	**1,271€**<br>[786 – 1,756]<br>*118*	*0,7*	**1,063€**<br>[735 – 1,390]<br>*99*	**2,673€**<br>[778 – 4,567]<br>*249*	*0,06*	**1,074€**<br>[813 – 1,334]<br>*100*	**2,425€**<br>[725 - 4,124]<br>*226*	*0.08*
	**IADL**	**1,339€**<br>[1,051 - 1,628]<br>*125*	**1,569€**<br>[1,117 – 2,021]<br>*146*	*0,4*	**1,135€**<br>[927 – 1,344]<br>*106*	**1,189€**<br>[692 – 1,686]<br>*111*	*0,8*	**1,005€**<br>[815 – 1,195]<br>*94*	**1,641€**<br>[738 – 2,544]<br>*153*	*0,2*	**1,200€**<br>[930 - 1470]<br>*112*	**1,430€**<br>[751 – 2,110]<br>*133*	*0,2*
	**Supervision**	**8,086€**<br>[6,918 – 9,254]<br>*754*	**8,505€**<br>[6,675 – 10,336]<br>*793*	*0,7*	**8,924€**<br>[7,738 – 10,110]<br>*832*	**8,528€**<br>[6,574 – 10,482]<br>*795*	*0,7*	**8,039€**<br>[6,712 – 9,367]<br>*750*	**7,451€**<br>[5,382 – 9,520]<br>*694*	*0,1*	**9,131€**<br>[7,705 – 10,556]<br>*851*	**9,940€**<br>[7,838 – 12,041]<br>*926*	*> 0,9*
Total w/ supervision		10,380€<br>[9,028 – 11,733]	11,562€<br>[9,466 – 13,658]	*0,3*	11,235€<br>[9,876 – 12,593]	10,988€<br>[8,710 – 13,267]	*0,8*	10,107€<br>[8,537 – 11,677]	14,249€<br>[10,455 – 18,043]	***0.04***	11,405€<br>[9,762 – 13,049]	13,969€<br>[10,611 – 17,326]	*0.1*
Total w/o supervision		2,294€<br>[1,777 – 2,810]	3,056€<br>[2,145 – 3,967]	*0,1*	2,310€<br>[1,748 – 2,792]	2,460€<br>[1,573 – 3,347]	*0,7*	2,068€<br>[1,609 – 2,526]	4,314€<br>[1,632 – 6,995]	*0,1*	2,274€<br>[1,822 – 2,726]	4,029€<br>[1,714 – 6,344]	*0,2*
**Medications**<br>[IC 95%]	**Alzheimer's disease**	**570€**<br>[566 - 575]	**571€**<br>[566 - 575]	*0,6*	**526€**<br>[521 - 530]	**531€**<br>[526 - 536]	*0,5*	**533€**<br>[528 - 538]	**533€**<br>[527 - 540]	*> 0,9*	**528€**<br>[525 - 532]	**526€**<br>[520 - 532]	*0,4*
	**Psychotropic**	**44€**<br>[28 - 59]	**41€**<br>[20 - 63]	*0,8*	**41€**<br>[30 - 52]	**55€**<br>[24 - 85]	*0,4*	**38€**<br>[26 - 49]	**46€**<br>[18 - 74]	*0,6*	**80€**<br>[16 - 144]	**133€**<br>[26 - 239]	*0,4*
Total		583€<br>[575 - 590]	584€<br>[571 – 597]	*0,2*	538€<br>[532 – 545]	554€<br>[534 - 570]	*0.06*	547€<br>[540 – 554]	550€<br>[534 – 566]	*0,7*	549€<br>[532 – 567]	570€<br>[531 – 610]	*0,3*

Supplementary material

e-Table 1 (continued): Trimestral €2022 costs according to the place of living of the patient and to the NPI-C clusters, Community dwelling patients, n = 183

		Mean costs per patient T1<br>n = 157			Mean costs per patient T2<br>n = 146			Mean costs per patient T3<br>n = 137			Mean costs per patient T4<br>n = 125		
		**Group A**<br>**n = 113**	**Group B**<br>**n = 44**	*p*	**Group A**<br>**n = 109**	**Group B**<br>**n = 37**	*p*	**Group A**<br>**n = 104**	**Group B**<br>**n = 33**	*p*	**Group A**<br>**n = 92**	**Group B**<br>**n = 33**	*p*
**Formal Care**<br>[IC 95%]<br>*Nbr H µ*	**Caregiver**	**206€**<br>[36 - 376]<br>*14*	**159€**<br>[6 - 312]<br>*11*	*0,7*	**132€**<br>[39 - 225]<br>*9*	**91€**<br>[31 - 214]<br>*6*	*0,6*	**138€**<br>[53 - 223]<br>*9*	**159€**<br>[13 - 331]<br>*11*	*0,8*	**205€**<br>[89 - 321]<br>*14*	**167€**<br>[4 - 340]<br>*11*	*0,7*
	**Home Care**	**512€**<br>[131 - 892]<br>*48*	**127€**<br>[26 - 228]<br>*12*	***0,05***	**532€**<br>[93 - 972]<br>*50*	**98€**<br>[26 - 170]<br>*9*	***0,05***	**468€**<br>[29 - 907]<br>*44*	**95€**<br>[5 - 195]<br>*9*	*0,1*	**584€**<br>[133 – 1,035]<br>*54*	**105€**<br>[35 - 246]<br>*10*	***0,04***
	**Day-care center**	**84€**<br>[28 - 140]<br>*15*	**114€**<br>[6 - 223]<br>*20*	*0,6*	**114€**<br>[41 - 187]<br>*20*	**96€**<br>[10 - 203]<br>*17*	*0,1*	**98€**<br>[28 - 168]<br>*17*	**179€**<br>[45 - 314]<br>*32*	*0,3*	**95€**<br>[35 - 154]<br>*17*	**165€**<br>[31 - 300]<br>*29*	*0,3*
	**Social worker**	**2€**<br>[0 - 5]<br>*0,1*	**2€**<br>[1 - 5]<br>*0,1*	*0,8*	**0€**<br>/<br>*0*	**0€**<br>/<br>*0*		**0€**<br>/<br>*0*	**1€**<br>[0 - 3]<br>*< 0,1*	*0,3*	**1€**<br>[1 - 3]<br>*< 0,1*	**1€**<br>[0 – 3]<br>*< 0,1*	*0,1*
	**Teleassistance**	**9€**<br>[3 - 16]	**11€**<br>[0 - 22]	*0,7*	**8€**<br>[2 - 15]	**8€**<br>[1 - 18]	*> 0,9*	**5€**<br>[0 - 9]	**9€**<br>[1 - 19]	*0,5*	**7€**<br>[1 - 12]	**9€**<br>[1 - 20]	*0,7*
[IC 95%]<br>*Nbr of users µ*	**Meals on wheels**	**217€**<br>[93 - 341]	**51€**<br>[1 - 153]	***0,04***	**158€**<br>[51 - 265]	**101€**<br>[41 - 244]	*0,5*	**193€**<br>[70 - 315]	**135€**<br>[56 - 326]	*0,6*	**171€**<br>[55 - 287]	**68€**<br>[7 - 205]	*0,2*
	Total	1,000€<br>[551 – 1,448]	453€<br>[218 – 687]	***0,03***	743€<br>[363 – 1,123]	356€<br>[137 – 575]	*0,1*	701€<br>[328 – 1,074]	492€<br>[190 – 794]	*0,4*	829€<br>[443 – 1,214]	447€<br>[185 – 709]	*0,1*

Supplementary material

e-Table 1 (continued): Trimestral €2022 costs according to the place of living of the patient and to the NPI-C clusters, Community dwelling patients, n = 183

		Mean costs per patient T1<br>n = 157		Mean costs per patient T2<br>n = 146		Mean costs per patient T3<br>n = 137		Mean costs per patient T4<br>n = 125	
		n = 113	n = 44	n = 109	n = 37	n = 104	n = 33	n = 92	n = 33
**Hospitalizations**<br>Nbr of stays	Psychiatry	Group A<br>n = 1<br>3,841€	Group B<br>n = 0<br>0€	Group A<br>n = 0<br>0€	Group B<br>n = 0<br>0€	Group A<br>n = 0<br>0€	Group B<br>n = 0<br>0€	Group A<br>n = 0<br>0€	Group B<br>n = 1<br>3,841€
	Geriatric	Group A<br>n = 46<br>4,227€	Group B<br>n = 11<br>4,383€	Group A<br>n = 33<br>4,275€	Group B<br>n = 9<br>3,985€	Group A<br>n = 15<br>3,427€	Group B<br>n = 1<br>4,383€	Group A<br>n = 2<br>4,383€	Group B<br>n = 9<br>3,958€
	Internal Medecine	Group A<br>n = 1<br>5,014€	Group B<br>n = 0<br>0€	Group A<br>n = 3<br>5,014€	Group B<br>n = 1<br>5,014€	Group A<br>n = 5<br>5,014€	Group B<br>n = 1<br>5,014€	Group A<br>n = 1<br>5,014€	Group B<br>n = 0<br>0€
	Surgery	Group A<br>n = 4<br>4,122€	Group B<br>n = 0<br>0€	Group A<br>n = 4<br>4,122€	Group B<br>n = 1<br>2,050€	Group A<br>n = 4<br>8,267€	Group B<br>n = 1<br>2,050€	Group A<br>n = 4<br>4,122€	Group B<br>n = 1<br>10,339€
	Others	Group A<br>n = 14<br>2,691€	Group B<br>n = 3<br>2,936€	Group A<br>n = 39<br>2,626€	Group B<br>n = 6<br>1,835€	Group A<br>n = 10<br>2,953€	Group B<br>n = 2<br>2,385€	Group A<br>n = 10<br>1,800€	Group B<br>n = 3<br>1,835€

Total	2,279€	1,296€	2,536€	1,458€	1,337€	491€	525€	1,683€

Supplementary material

e-Table 1 (continued): Trimestral €2022 costs according to the place of living of the patient and to the NPI-C clusters

Community dwelling patients, n = 183

		**Mean costs per patient T1**<br>**n = 157**			**Mean costs per patient T2**<br>**n = 146**			**Mean costs per patient T3**<br>**n = 137**			**Mean costs per patient T4**<br>**n = 125**		
		**Group A**<br>**n = 113**	**Group B**<br>**n = 44**	*p*	**Group A**<br>**n = 109**	**Group B**<br>**n = 37**	*p*	**Group A**<br>**n = 104**	**Group B**<br>**n = 33**	*p*	**Group A**<br>**n = 92**	**Group B**<br>**n = 33**	*p*
**Consultations**<br>[IC 95%]<br>*Nbr consultations µ*	General Practice	**69€**<br>[64 - 74]<br>*2,5*	**78€**<br>[62 - 94]<br>*3*	*0,3*	**59€**<br>[49 - 68]<br>*2*	**59€**<br>[45 - 74]<br>*2*	*> 0,9*	**57€**<br>[46 - 67]<br>*2*	**83€**<br>[4 - 143]<br>*3*	*0,4*	**81€**<br>[63 - 99]<br>*3*	**57€**<br>[44 - 71]<br>*2*	*0,5*
	Geriatric Medecine	**83€**<br>[60 - 105]<br>*3*	**76€**<br>[32 - 120]<br>*3*	*0,8*	**26€**<br>[18 - 33]<br>*1*	**30€**<br>[17 - 42]<br>*1*	*0,6*	**64€**<br>[20 - 108]<br>*2*	**30€**<br>[12 - 47]<br>*1*	*0,1*	**35€**<br>[10 - 61]<br>*1*	**96€**<br>[16 - 208]<br>*4*	*0,3*
	Neurology	**3€**<br>[0 - 8]<br>*0,1*	**0€**<br>/<br>*/*		**0€**<br>/<br>*/*	**0€**<br>/<br>*/*	*/*	**2€**<br>[0 - 6]<br>*0,1*	**7€**<br>[0 - 21]<br>*0,2*	*0,5*	**0€**<br>/<br>*/*	**0€**<br>/<br>*/*	
	Psychiatry	**3€**<br>[0 - 8]<br>*0,1*	**3€**<br>[0 - 10]<br>*0,1*	*0,9*	**0€**<br>/<br>*/*	**3€**<br>[1 - 10]<br>*0,1*		**1€**<br>[0 - 4]<br>*0,1*	**0€**<br>/<br>*/*	*/*	**0€**<br>/<br>*/*	**3€**<br>[1 - 10]<br>*0,1*	*/*
	Total	152€<br>[127 - 175]	157€<br>[100 – 214]	*> 0,9*	88€<br>[74 - 102]	96€<br>[72 - 120]	*0,5*	121€<br>[76 - 166]	116€<br>[55 - 177]	*> 0,9*	109€<br>[77 - 140]	171€<br>[54 - 288]	*0,3*

Supplementary material

e-Table 1 (continued): Trimestral €2022 costs according to the place of living of the patient and to the NPI-C clusters

Community dwelling patients, n = 183

		Mean costs per patient T1<br>n = 157			Mean costs per patient T2<br>n = 146			Mean costs per patient T3<br>n = 137			Mean costs per patient T4<br>n = 125		
		**Group A**<br>**n = 113**	**Group B**<br>**n = 44**	*p*	**Group A**<br>**n = 109**	**Group B**<br>**n = 37**	*p*	**Group A**<br>**n = 104**	**Group B**<br>**n = 33**	*p*	**Group A**<br>**n = 92**	**Group B**<br>**n = 33**	*p*
**Paramedical Acts**<br>[IC 95%]<br>*Nbr consultations μ*<br>[IC 95%]<br>*Nbr H μ*	Physical therapy	**144€**<br>[89 - 198]<br>*7*	**141€**<br>[42 - 241]<br>*7*	*> 0,9*	**171€**<br>[115 - 228]<br>*7*	**68€**<br>[3 - 132]<br>*3*	***0,01***	**149€**<br>[89 - 208]<br>*7*	**110€**<br>[19 - 202]<br>*4*	*0,5*	**191€**<br>[107 - 275]<br>*9*	**90€**<br>[28 - 152]<br>*4*	***0,05***
	Ergotherapy	**4€**<br>[0 - 13]<br>*0,1*	**9€**<br>[0 - 21]<br>*0,1*	*0,5*	**4€**<br>[0 - 13]<br>*0,1*	**0€**<br>/<br>*/*	*/*	**17€**<br>[6 - 50]<br>*0,2*	**18€**<br>[8 - 54]<br>*0,3*	*> 0,9*	**30€**<br>[7 - 66]<br>*0,4*	**27€**<br>[13 - 66]<br>*0,4*	*0,7*
	Practice Nurse	**1,217€**<br>[899 – 1,535]<br>*47*	**744€**<br>[410 – 1,077]<br>*38*	***0,04***	**1,453€**<br>[1,074 – 1,832]<br>*65*	**1,462€**<br>[693 – 2,232]<br>*57*	*> 0,9*	**1,992€**<br>[1,476 – 2,508]<br>*78*	**1,659€**<br>[960 – 2,358]<br>*70*	*0,4*	**1,856€**<br>[1,386 – 2,326]<br>*77*	**1,960€**<br>[1,085 – 2,835]<br>*85*	*0,9*
Total		1,365€<br>[1,027 – 1,702]	894€<br>[546 – 1,242]	0.05	1,629€<br>[1,221 – 2,037]	1,530€<br>[761 – 2,299]	0.82	2,158€<br>[1,616 – 2,700]	1,787€<br>[1,074 – 2,500]	0.4	2,076€<br>[1,562 – 2,590]	2,077€<br>[1,175 – 2,979]	> 0,9
**Nursing Home costs**		/	/		/	/		/	/		/	/	
**Global costs – w/o sup.**		**7,673€**	**6,441€**		**7,845€**	**6,454€**		**6,932€**	**10,234€**		**6,362€**	**8,977€**	
**Global costs – w/ sup.**		**15,759€**	**14,946€**		**16,769€**	**14,982€**		**14,971€**	**17,685€**		**15,493€**	**18,917€**	

Supplementary material

e-Table 2: Trimestral €2022 costs according to the place of living of the patient and to the NPI-C clusters
Nursing home patients, n = 79

		Mean costs per patient T1<br>n = 71			Mean costs per patient T2<br>n = 66			Mean costs per patient T3<br>n = 57			Mean costs per patient T4<br>n = 51		
		Group A<br>n = 48	Group B<br>n = 23	*p*	Group A<br>n = 45	Group B<br>n = 21	*p*	Group A<br>n = 38	Group B<br>n = 19	*p*	Group A<br>n = 33	Group B<br>n = 18	*p*
**Informal Care**<br>[IC 95%]<br>*Nbr H µ*	**ADL**	**/**			**/**			**/**			**/**		
	**IADL**	**/**			**/**			**/**			**/**		
	**Supervision**	**/**			**/**			**/**			**/**		
Total		**/**			**/**			**/**			**/**		
		**Group A**<br>**n = 46**	**Group B**<br>**n = 20**	*p*	**Group A**<br>**n = 43**	**Group B**<br>**n = 18**	*p*	**Group A**<br>**n = 36**	**Group B**<br>**n = 16**	*p*	**Group A**<br>**n = 32**	**Group B**<br>**n = 15**	*p*
	**Alzheimer's disease**	**574€**<br>[568 - 579]	**574€**<br>/<br>*/*		**526€**<br>[522 – 531]	**522€**<br>[508 - 536]<br>*p = 0,5*		**529€**<br>/	**529€**<br>/		**529€**<br>/	**529€**<br>/<br>*/*	
		**Group A**<br>**n = 15**	**Group B**<br>**n = 9**	*p*	**Group A**<br>**n = 13**	**Group B**<br>**n = 9**	*p*	**Group A**<br>**n = 7**	**Group B**<br>**n = 7**	*p*	**Group A**<br>**n = 6**	**Group B**<br>**n = 7**	*p*
**Medications**<br>[IC 95%]	**Psychotropic**	**80€**<br>[1 - 159]	**36€**<br>[5 – 77]	*0,3*	**103€**<br>[71 - 279]	**69€**<br>[10 - 148]	*0,6*	**42€**<br>[15 - 69]	**32€**<br>[1 - 64]	*0,5*	**76€**<br>[4 - 554]	**43€**<br>[2 - 116]	*0,5*
Total		600€<br>[573 – 627]	590€<br>[572 – 609]	*0,5*	460€<br>[400 – 520]	487€<br>[408 – 567]	*0,5*	395€<br>[324 - 465]	431€<br>[327 – 535]	*0,6*	371€<br>[297 - 445]	403€<br>[291 – 515]	*0,6*

e-Table 2 (continued): Trimestral €2022 costs according to the place of living of the patient and to the NPI-C clusters, Nursing home patients, n = 79

		**Mean costs per patient T1**<br>**n = 71**			**Mean costs per patient T2**<br>**n = 66**			**Mean costs per patient T3**<br>**n = 57**			**Mean costs per patient T4**<br>**n = 51**		
		**Group A**<br>**n = 48**	**Group B**<br>**n = 23**	*p*	**Group A**<br>**n = 45**	**Group B**<br>**n = 21**	*p*	**Group A**<br>**n = 38**	**Group B**<br>**n = 19**	*p*	**Group A**<br>**n = 33**	**Group B**<br>**n = 18**	*p*
**Formal Care**<br>[IC 95%]<br>*Nbr H µ*	**Caregiver**	/			/			/			/		
	**Home Care**	/			/			/			/		
	**Day-care center**	/			/			/			/		
	**Social worker**	/			/			/			/		
[IC 95%]<br>*Nbr of users µ*	**Teleassistance**	/			/			/			/		
	**Meals on wheels**	/			/			/			/		
Total		/			/			/			/		

e-Table 2 (continued): Trimestral €2022 costs according to the place of living of the patient and to the NPI-C clusters, Nursing home patients, n = 79

		Mean costs per patient T1 n = 71		Mean costs per patient T2 n = 66		Mean costs per patient T3 n = 57		Mean costs per patient T4 n = 51	
		n = 48	n = 23	n = 45	n = 21	n = 38	n = 19	n = 33	n = 18
**Hospitalizations** Nbr of stays	**Psychiatry**	**Group A n = 0** **0€**	**Group B n = 0** **0€**	**Group A n = 0** **0€**	**Group B n = 0** **0€**	**Group A n = 0** **0€**	**Group B n = 0** **0€**	**Group A n = 0** **0€**	**Group B n = 0** **0€**
	**Geriatric**	**Group A n = 19** **4,383€**	**Group B n = 12** **4,383€**	**Group A n = 4** **3,487€**	**Group B n = 2** **2,590€**	**Group A n = 1** **4,383€**	**Group B n = 1** **4,383€**	**Group A n = 3** **4,383€**	**Group B n = 1** **4,383€**
	**Internal Medecine**	**Group A n = 1** **5,014€**	**Group B n = 0** **0€**	**Group A n = 1** **5,014€**	**Group B n = 0** **0€**	**Group A n = 0** **0€**	**Group B n = 0** **0€**	**Group A n = 0** **0€**	**Group B n = 0** **0€**
	**Surgery**	**Group A n = 0** **0€**	**Group B n = 0** **0€**	**Group A n = 5** **10,339€**	**Group B n = 2** **15,421€**	**Group A n = 0** **0€**	**Group B n = 0** **0€**	**Group A n = 1** **10,339€**	**Group B n = 1** **2,050€**
	**Others**	**Group A n = 4** **2,385€**	**Group B n = 2** **734€**	**Group A n = 8** **1,195€**	**Group B n = 6** **2,095€**	**Group A n = 2** **734€**	**Group B n = 1** **734€**	**Group A n = 1** **734€**	**Group B n = 0** **0€**
Total		1,764€	1,978€	2,306€	2,051€	153€	269€	733€	327€

Supplementary material

e-Table 2 (continued): Trimestral €2022 costs according to the place of living of the patient and to the NPI-C clusters, Nursing home patients, n = 79

		**Mean costs per patient T1**<br>**n = 71**			**Mean costs per patient T2**<br>**n = 66**			**Mean costs per patient T3**<br>**n = 57**			**Mean costs per patient T4**<br>**n = 51**		
		**Group A**<br>**n = 48**	**Group B**<br>**n = 23**	*p*	**Group A**<br>**n = 45**	**Group B**<br>**n = 21**	*p*	**Group A**<br>**n = 38**	**Group B**<br>**n = 19**	*p*	**Group A**<br>**n = 33**	**Group B**<br>**n = 18**	*p*
**Consultations**<br>[IC 95%]<br>*Nbr consultations µ*	General Practice	**52€**<br>[33 - 72]<br>*2,2*	**127€**<br>[14 - 241]<br>*4,7*	*0,2*	**46€**<br>[29 - 63]<br>*1,4*	**32€**<br>[14 - 49]<br>*1,2*	*0,2*	**47€**<br>[15 – 79]<br>*1,5*	**46€**<br>[11 - 80]<br>*1,7*	*> 0,9*	**36€**<br>[20 - 51]<br>*1,1*	**64€**<br>[24 - 103]<br>*2,3*	*0,2*
	Geriatric Medecine	**114€**<br>[64 - 163]<br>*4,0*	**173€**<br>[89 - 258]<br>*6,4*	*0,2*	**52€**<br>[13 - 92]<br>*1,5*	**159€**<br>[72 - 245]<br>*5,3*	***0,02***	**34€**<br>[14 - 54]<br>*0,8*	**133€**<br>[25 - 240]<br>*4,0*	*0,07*	**24€**<br>[14 - 43]<br>*0,6*	**94€**<br>[4 - 184]<br>*2,9*	*0,1*
	Neurology	**0€**<br>/<br>*/*	**13€**<br>[4 - 41]<br>*0,3*	*/*	**0€**<br>/<br>*/*	**13€**<br>[6 - 45]<br>*0,3*	*/*	**3€**<br>[0 - 10]<br>*0,05*	**0€**<br>/<br>*/*		**0€**<br>/<br>*/*	**0€**<br>/<br>*/*	
	Psychiatry	**0€**<br>/<br>*/*	**0€**<br>/<br>*/*		**0€**<br>/<br>*/*	**0€**<br>/<br>*/*		**3€**<br>[0 - 9]<br>*0,05*	**0€**<br>/<br>*/*		**0€**<br>/<br>*/*	**0€**<br>/<br>*/*	
Total		165€<br>[120 - 212]	314€<br>[173 - 455]	*0,04*	81€<br>[46 - 116]	190€<br>[87 - 293]	*0,04*	69€<br>[37 - 102]	156€<br>[67 - 244]	*0,06*	46€<br>[27 - 66]	142€<br>[64 - 219]	*0,02*

Supplementary material

e-Table 2 (continued): Trimestral €2022 costs according to the place of living of the patient and to the NPI-C clusters, Nursing home patients, n = 79

		Mean costs per patient T1<br>n = 71			Mean costs per patient T2<br>n = 66			Mean costs per patient T3<br>n = 57			Mean costs per patient T4<br>n = 51		
		**Group A**<br>**n = 48**	**Group B**<br>**n = 23**	*p*	**Group A**<br>**n = 45**	**Group B**<br>**n = 21**	*p*	**Group A**<br>**n = 38**	**Group B**<br>**n = 19**	*p*	**Group A**<br>**n = 33**	**Group B**<br>**n = 18**	*p*
**Paramedical Acts**<br>[IC 95%]<br>*Nbr consultations µ*	Physical therapy	**116€**<br>[15 - 216]<br>*7*	**88€**<br>[43 - 220]<br>*3,9*	*0,7*	**126€**<br>[29 - 223]<br>*5,7*	**104€**<br>[41 - 249]<br>*4,7*	*0,8*	**90€**<br>[14 - 166]<br>*3,5*	**83€**<br>[31 - 197]<br>*4,5*	*> 0,9*	**93€**<br>[20 - 165]<br>*4,2*	**87€**<br>[1 - 174]<br>*4,5*	*> 0,9*
	Ergotherapy	**12€**<br>[2 - 37]<br>*0,2*	**68€**<br>[3 - 209]<br>*1,0*	*0,4*	**49€**<br>[9 - 147]<br>*0,6*	**42€**<br>[5 - 130]<br>*0,6*	*> 0,9*	**49€**<br>[9 - 147]<br>*0,6*	**68€**<br>[3 - 209]<br>*1,0*	*0,8*	**37€**<br>[7 - 110]<br>*0,3*	**68€**<br>[9 - 165]<br>*1,0*	*0,6*
Total		197€<br>[60 - 333]	124€<br>[41 - 287]	*0,5*	176€<br>[46 - 307]	133€<br>[6 - 273]	*0,6*	104€<br>[10 - 198]	246€<br>[32 - 524]	*0,3*	107€<br>[24 - 190]	120€<br>[0 - 239]	*0,8*
**Nursing Home costs**		11,144€	11,144€		11,144€	11,144€		11,144€	11,144€		11,144€	11,144€	
**Global costs**		**13,870€**	**14,150€**		**14,167€**	**14,005€**		**11,865€**	**12,017€**		**12,401€**	**12,136€**	
